# Distribution of water turnover by sex and age as estimated by prediction equation in Japanese adolescents and adults: the 2016 National Health and Nutrition Survey, Japan

**DOI:** 10.1186/s12937-023-00896-z

**Published:** 2023-11-29

**Authors:** Daiki Watanabe, Yumiko Inoue, Motohiko Miyachi

**Affiliations:** 1 Faculty of Sport Sciences, Waseda University, 2-579-15 Mikajima, Tokorozawa-city, Saitama 359-1192 Japan; 2 National Institute of Health and Nutrition, National Institutes of Biomedical Innovation, Health and Nutrition, 17-34 Senrioka-Shimmachi, Settsu-city, Osaka 566-0002 Japan; 3 Graduate School of Sport Sciences, Waseda University, 2-579-15 Mikajima, Tokorozawa-city, Saitama 359-1192 Japan

**Keywords:** Standard values, Water requirement, Self-reported dietary assessment, Biomarker, Dose-response relationship

## Abstract

**Background:**

Although water is essential to the maintenance of health and life, standard values for human water requirements are yet to be determined. This study aimed to evaluate the distribution of water turnover (WT) according to sex and age, estimated using a prediction equation, in Japanese adolescents and adults.

**Methods:**

This cross-sectional study used data from the 2016 National Health and Nutrition Survey, Japan. Data were obtained from electronically available aggregated reports in the survey’s official website. Participants aged between 15 and 80 years (10,546 men, 12,355 women) were selected using stratified random sampling. WT was calculated considering lifestyle and environmental factors, and using an equation (coefficient of determination = 0.471) previously developed by the international doubly labelled water (DLW) database group. As data on physical activity levels (PAL) were not collected in the survey, we used two evaluation methods: (1) energy intake assessed by dietary records and (2) total energy expenditure measured by the DLW method reported in previous Japanese studies, divided by basal metabolic rate predicted using the equation. We evaluated the relationship between WT and age using a restricted cubic spline model.

**Results:**

The average WT for the 15–19, 20–29, 30–39, 40–49, 50–59, 60–69, and ≥ 70 years was 3291, 3151, 3213, 3243, 3205, 3104, and 2790 ml/day, respectively in men, and 2641, 2594, 2741, 2739, 2753, 2707, and 2482 ml/day, respectively in women. In the spline model, WT showed an inverse association with age in men older than 50 years, whereas women showed a reverse U-shaped relationship between WT and age (*p* for non-linearity < 0.001), although results differed with body weight adjustment. Similar results were found for both PAL evaluation methods, and the range of WT per body weight was 45–56 ml/day for both sexes.

**Conclusions:**

We determined the standard values of WT in Japanese population using a prediction equation and national large-scale survey data. These findings may be useful for setting water requirements for dietary guidelines in future.

**Supplementary Information:**

The online version contains supplementary material available at 10.1186/s12937-023-00896-z.

## Background

When the fluid balance of the body is disturbed by the loss of water, humans experience hunger or thirst, and adjust primarily by consuming water through food and beverages [1]. Homeostatic functions maintain fluid levels in the body [2]. Although humans are thought to have evolved to utilise less water per day compared to other primates [3], a few days spent without consuming water can prove fatal to them [4, 5]. Therefore, it is important to evaluate the amount of water required for maintaining life and good health.

The requirement of water consumption as per guidelines from the United States and Canada [6] and the World Health Organisation (WHO) [7] are 2.7 L/day for adult women and 3.7 L/day for adult men. However, the Dietary Reference Intake in Japan does not indicate set water requirements for Japanese people [8]. Regarded as a gold standard method to evaluate the required amount of daily water consumption, water turnover (WT) can be measured using the deuterium or the double-labelled water method [3, 4, 9–11]. WT has been shown to differ between countries and regions with different environments [1, 4]. The sources of human water inputs are metabolic water produced by the metabolism of nutrients, respiratory and transcutaneous water entering the body through breathing and the skin, and pre-formed water derived from ingested food and fluids [9]. Therefore, clarifying the requirement of WT and pre-formed water in individuals is essential for establishing recommendations and setting goal values for water consumption to prevent dehydration and maintain body fluids [12].

To determine the water requirements for guidelines, standard values should be established by evaluating the mean and distributions of WT measured by DLW methods according to sex and age from a randomly selected sample population of Japanese individuals [8]. However, it is difficult to obtain such data because the DLW method is an expensive experimental technique. Intake values estimated from dietary records (DRs) are often underestimated [13–15], making it difficult to accurately estimate water as well as energy intake (EI) [11]. Recently, the international DLW database group, in which we are a participant, developed a prediction equation for WT measured by DLW methods [4]. This prediction equation included significant factors such as anthropometric, lifestyle, and environmental factors, and can obtain predicted WT for human in different countries and regions around the world. However, to the best of our knowledge, standard values of WT have not been considered, and we believe that it may be possible to estimate standard values for the Japanese population using this equation and national large-scale survey data. Thus, this study aims to estimate the expected distribution of WT by age and sex in Japanese adults based on inputs for the previously developed prediction equation [4] and lifestyle and environmental factors. In addition, we compare the distribution of pre-formed water estimated by the prediction equation with DRs. This study aims to obtain useful knowledge for setting standard values of WT using a prediction equation and national large-scale survey data. Further, we hypothesised that pre-formed-water estimated from DR was underestimated compared with those estimated by prediction equation.

## Methods

### Study design and data

This study was conducted in the following steps: (1) data were collected from electronically available aggregated reports; (2) WT was estimated according to age and sex from the collected data using the previously developed prediction equation, and (3) pre-formed water calculated by subtracting metabolic, respiratory, transcutaneous derived water from predicted WT was compared with the value estimated from DRs.

We used data from the 2016 National Health and Nutrition Survey, Japan (NHNS-J) [16–18]. This survey commenced in 1946, with the purpose of obtaining basic data necessary to receive food aid after World War II from each country under the General Headquarters of the Allied Forces. It is a cross-sectional household examination survey conducted in November of each year with the exception of 2016, when it was conducted from 1 October to 30 November because of the exceptionally large sample. Participants were selected using stratified random sampling from the 2010 census enumeration area of all 47 prefectures of Japan, with 10 districts per prefecture (only Tokyo, which has a large population, has 15 districts). In these areas, household members aged one year or older were included, except for the 13 districts affected by natural disasters, such as earthquakes and typhoons, in 2016. The number of households that gave valid responses to the dietary section of this survey was 10,745 out of 24,187 households (response rate = 44.4%). The present analyses included individuals aged 15–80 years who completed the dietary survey (10,546 men and 12,355 women).

Dietary intake was assessed from single-day semi-weighed household DRs, excluding trip or festivity days [19]. Before the dietary survey, participants were instructed on how to complete the DR by well-trained workers (mainly registered dietitians). A household member, usually responsible for preparing meals, was entrusted with weighing all food and beverages consumed by the household members and assigning approximate proportions of each dietary item to individual household members. When data were missing or illogical, workers visited the household and verified the portion sizes of food and beverages on the DR forms. Energy and nutrient intake were calculated from the weight of food and beverages consumed, based on the 2010 Standard Tables of Food Composition in Japan [20]. After the participants had completed the dietary survey, a physical examination was conducted in a facility within walking distance of their residence. The height and body weight were evaluated to the nearest 0.1 cm and 0.1 kg, respectively, using a stadiometer and weighing scale, with the participants barefoot and wearing light clothing. If participants were unavailable to measure these variables or could not travel to the facility, values were obtained from self-reports or measured at their homes. All data were available electronically in the form of aggregated reports in the official website of the Ministry of Health, Labour, and Welfare [21].

### Estimation of WT

The WT was calculated using a previously developed equation derived from select variables using a multiple regression model with the WT measured by DLW methods as a dependent variable [4]. Data were collected from 5604 people including Japanese data that we provided for the ages of eight days to 96 years from across 23 countries and stored in the international DLW database, based on which a model predictive equation for WT in adults aged 18 years and older was developed. The coefficient of determination (R^2^) for this model was 0.471. The model used the following equation [4]:

1 $$\begin{aligned}C & = {\beta _0} + {\beta _1}\;PA{L_1} + {\beta _2}\;body\; weigh{t_2}\\&\quad + {\beta _3}\;se{x_3} (1\;if\;men,\;0\;if\;women) \\ &\quad + {\beta _4}\;humidit{y_4}\\&\quad + {\beta _5}\;athlete\; statu{s_5} (1\;if\;yes,0\;if\;no) \\ & \quad + {\beta _6}\;HD{I_6}(2\;if\;low\;HDI,1\;if\;middle\;HDI,0\;if\;high\;HDI) \\&\quad + {\beta _7}\;altitud{e_7} + {\beta _8}\;ag{e^2}_8 + {\beta _9}\;ag{e_9}\\&\quad + {\beta _{10}}\;temperatur{e^2}_{10} + {\beta _{11}}\;temperatur{e_{11}} \end{aligned}$$

where *C* represents WT. The intercept (β_0_) of the equation was − 713.1 ml. The coefficients of the binary variables of sex (β_3_), athlete status (β_5_), and human development index (HDI; β_6_), were 374.9 ml, 1070 ml, and 104.6 ml, respectively. The coefficients of the continuous variables of physical activity level (PAL; β_1_), body weight (β_2_), humidity (β_4_), altitude (β_7_), age^2^ (β_8_), age (β_9_), temperature^2^ (β_10_), and temperature (β_11_), were 1076 ml, 14.34 ml (kg), 5.823 ml (%), 0.4726 ml (m), − 0.3529 ml (years), 24.78 ml (years), 1.865 ml (℃), and − 19.66 ml (℃), respectively. The product of the above coefficients and all characteristic variables of the participants, such as PAL (continuous), body weight (continuous), sex (1 for men, 0 for women), humidity (continuous), athlete status (1 for yes, 0 for no), HDI (2 for low HDI countries, 1 for middle HDI countries, 0 for high-HDI countries), altitude (continuous), age^2^ (continuous), age (continuous), temperature^2^ (continuous), and temperature (continuous) were calculated. WT was calculated as the sum of the above products and intercepts.

### Other variables

Calibrated EI was calculated by multiplying the EI estimated from the DR by 1.09, because it was previously reported that the values estimated from DRs were underestimated by approximately 9% compared with total energy expenditure (TEE), as measured using the DLW method in Japanese older adults [15]. The predictive basal metabolic rate (pBMR) was estimated using the equation by Ganpule et al. for Japanese individuals [22], given that this equation provided the best results in a comparison of several different equations for calculating pBMR [23]. We assumed that EI and TEE had similar values [24], and PAL was substituted with calibrated EI/pBMR to compensate for the absence of PAL data in NHNS-J. As water intake from food was not reported in NHNS-J, and the mean ratio of water from foods in the DRs of previous studies was 69% [25], water intake from food was estimated at 69% of the food weight from the DR. Based on the sum of water intake from food and beverages, pre-formed water was estimated. Japan was considered a high-HDI country in accordance with a previous study [4]. We were unable to evaluate athletic status in this survey, therefore, we assumed that there were no athletes across all age groups. For altitude, a value of 189.2 m was used for all participants, which is the average value for the inhabited area in Japan [26]. The temperature and relative humidity during the survey period adopted the mean values as on November 2016 in all surveyed areas determined from the database of the Japan Meteorological Agency (temperature: 10.9℃ and relative humidity: 74.7%) [27].

### Calculation of other water consumption

The carbon dioxide production rate (rCO_2_), metabolic, respiratory, transcutaneous, and pre-formed water were calculated using Eqs. 2–6. We assumed that EI and TEE showed similar values, and rCO_2_ (mg/day) was calculated using the calibrated EI (kcal/day), food quotient (FQ), and Eq. (2) referenced in a previous study [28]:

2 $$rC{O_2} = calibrated\,EI/(1.106 + (3.94/FQ))$$

We assumed that the respiratory quotient was equal to the FQ, which was calculated by multiplying the coefficients (protein: 0.8, fat: 0.7, carbohydrates: 1.0) and dietary protein, fat, and carbohydrate intake. Metabolic water (*Wmet*; ml/day) was calculated using Eq. (3) [9]:

3 $$\eqalign{{W_{met}} = & Calibrated{\mkern 1mu}\;EI \times (1/100,000) \cr & \left[ {{{0.119}_{\% fat}} + {{0.103}_{\% pro}} + {{0.150}_{\% carb}} + {{0.168}_{\% alc}}} \right] \cr}$$

The intake of fat (%fat), protein (%pro), carbohydrates (%carb), and alcohol (%alc) per calibrated EI, as estimated from the single-day DR, were multiplied by their coefficients and totalled. Metabolic water was estimated by multiplying the total value by the calibrated EI (kcal/day). Respiratory water (*W*_*res*_; ml/day) was calculated from the concentration of water in the atmosphere, estimated from the average air temperature and relative humidity during the period when the DR was made, using Eq. (4) [9]:

4 $${W_{res}} = \left[ {absolute\,humidity/1000} \right] \times 0.035rC{O_2}$$

The mean temperature, relative humidity, and absolute humidity during the study were 10.9 °C, 74.7%, and 7.45 g/m^3^ in November 2016, respectively [27]. For respiratory air volume, 3.5% of the inhaled air was assumed to be CO_2_ and was calculated from the rCO_2_ obtained using above equation. Transcutaneous water (*W*_*trans*_; ml/day) was calculated using Eq. (5) [9]:

5 $${W_{trans}} = \left[ {{{0.18}_{absolute\;humidity}}/21.7} \right] \times 0.5 \times BSA \times 1.44$$

The transdermal absorption rate per m^2^ of body surface area (BSA) in an atmosphere saturated with water vapor (21.7 mg/L) was 0.18 g/m^2^. The BSA (m^2^) was estimated using the Dubois equation [29]. As clothing reduces the rate of evaporation of moisture from the skin, the clothing coefficient was assumed to be 50%. Pre-formed water (*W*_*pre*_; L/day) was calculated by subtracting the metabolic, respiratory, and transcutaneous water from the WT using Eq. (6) [9]:

6 $${W}_{pre}= WT-\left[{W}_{met}+{W}_{res}+{W}_{trans}\right]$$

Pre-formed water includes the fluids consumed from food and drinks.

### Statistical analysis

All analyses were performed after stratifying by age (15–19, 20–29, 30–39, 40–49, 50–59, 60–69, and ≥ 70 years) and sex (male or female) according to the summary tables reported by the NHNS-J. For descriptive statistics, continuous and categorical variables of participant characteristics were expressed as mean and standard deviations or 95% confidence intervals (CI), and as numbers and percentages, respectively.

We used a restricted cubic spline model with three knots based on age distribution (5th (18 years), 50th (48 years), and 95th (77 years) percentiles) to evaluate the curvilinearity of the relationship between water consumption and age [30, 31]. The statistical significance of non-linearity was assessed using the Wald test, comparing the likelihood ratio of the spline model with the linear model, with *p* values of < 0.05 indicating a statistically significant non-linear relationship between the WT and age [32]. For the sensitivity analysis, we performed the same procedure using the TEE evaluated by DLW methods reported in previous Japanese studies [15, 33–35] because EI from DRs may not be uniformly underestimated in age groups [13, 14].

We compared the distribution of pre-formed water estimated by the prediction equation and DRs according to sex- and age-stratified models. The analysis results are presented as mean differences and 95% CI. To assess the relationship between underestimation of pre-formed water estimated from the DR and age, the *p*-value of the linear trend was calculated using a regression model and the continuous variable of age.

A two-tailed significance level of 5% was adopted and STATA MP, version 15.0 (StataCorp LP, College Station, TX, USA) was used for all analyses.

## Results

Table 1 shows the participant characteristics stratified by age and sex in the analysed population. The number of participants increased with age in both sexes. Body weight was the highest for those aged 40–49 years, followed by an age-dependent decline up to ≥ 70 years in both sexes. The calibrated EI/pBMR tended to be high in the age ranges of 15–19 and ≥ 60 years. Comparison with the TEE reported in Japanese previous studies, calibrated EI took lower values, from approximately 3.5 (50–59 years in women) to 14.1% (30–39 years in men) but EI (uncalibration) took lower values, from approximately 11.5 (50–59 years in women) to 21.2% (30–39 years in men; Supplementary Table 1).

**Table 1:** Participant characteristics stratified by age and sex

	Participants by age group													
	15–19 years		20–29 years		30–39 years		40–49 years		50–59 years		60–69 years		≥ 70 years	
**Men**, ***n***	559		710		1207		1581		1486		2307		2696	
Height [cm]	170.1	(5.2)	171.5	(5.8)	171.5	(5.6)	171.4	(5.4)	169.8	(5.4)	167.0	(5.4)	162.1	(5.8)
Body weight [kg]	60.4	(9.2)	67.6	(12.4)	69.2	(11.1)	70.9	(11.4)	69.7	(10.0)	66.6	(9.0)	61.6	(9.0)
Body mass index [kg/m^2^]	20.9	(3.0)	22.9	(3.8)	23.5	(3.5)	24.1	(3.6)	24.1	(3.1)	23.9	(2.8)	23.4	(2.9)
Food and beverage weight [g/day]	2111	(645)	2029	(741)	2094	(689)	2192	(719)	2291	(697)	2397	(691)	2241	(614)
Fluid intake from beverages [ml/day]	1648	(N/A)	1620	(N/A)	1687	(N/A)	1775	(N/A)	1867	(N/A)	1945	(N/A)	1802	(N/A)
EI [kJ/day]	10,148	(2678)	8842	(2824)	8754	(2477)	8877	(2356)	8976	(2188)	9037	(2125)	8353	(1941)
Calibrated EI [kJ/day]	11,061	(2916)	9638	(3075)	9541	(2699)	9676	(2569)	9784	(2385)	9850	(2318)	9105	(2117)
Protein intake [g/day]	85.7	(26.4)	74.5	(26.2)	73.6	(22.7)	74.8	(22.9)	76.1	(21.6)	79.1	(22.4)	74.4	(20.9)
Fat intake [g/day]	76.6	(27.1)	67.7	(27.5)	65.0	(26.6)	64.6	(25.4)	63.2	(23.3)	61.7	(22.4)	54.7	(20.8)
Carbohydrate intake [g/day]	334.6	(99.4)	285.6	(107.5)	278.0	(86.7)	278.8	(83.3)	282.1	(76.6)	286.4	(75.2)	277.7	(70.4)
Food quotient	0.89	(N/A)	0.88	(N/A)	0.89	(N/A)	0.89	(N/A)	0.89	(N/A)	0.89	(N/A)	0.90	(N/A)
pBMR [kJ/day]	6237	(N/A)	6496	(N/A)	6439	(N/A)	6380	(N/A)	6147	(N/A)	5801	(N/A)	5368	(N/A)
Calibrated EI/pBMR	1.77	(N/A)	1.48	(N/A)	1.48	(N/A)	1.52	(N/A)	1.59	(N/A)	1.70	(N/A)	1.70	(N/A)
**Women**, ***n***	491		779		1350		1819		1777		2641		3498	
Height [cm]	157.0	(5.8)	158.2	(5.5)	158.1	(5.1)	157.9	(5.3)	156.7	(4.9)	153.4	(4.8)	148.8	(5.7)
Body weight [kg]	50.1	(6.6)	53.2	(8.8)	53.6	(8.4)	55.5	(9.8)	55.2	(8.7)	53.9	(7.8)	50.5	(8.2)
Body mass index [kg/m^2^]	20.3	(2.2)	20.9	(3.3)	21.5	(3.2)	22.3	(3.8)	22.5	(3.4)	22.9	(3.2)	22.8	(3.4)
Food and beverage weight [g/day]	1644	(508)	1646	(542)	1786	(569)	1831	(605)	1987	(536)	2073	(526)	1954	(559)
Fluid intake from beverages [ml/day]	1299	(N/A)	1312	(N/A)	1443	(N/A)	1494	(N/A)	1620	(N/A)	1680	(N/A)	1564	(N/A)
EI [kJ/day]	7420	(2033)	6823	(1895)	7087	(1866)	7017	(1828)	7225	(1669)	7324	(1615)	6957	(1699)
Calibrated EI [kJ/day]	8087	(2218)	7437	(2067)	7725	(2033)	7649	(1992)	7875	(1820)	7984	(1761)	7583	(1849)
Protein intake [g/day]	64.8	(19.8)	60.5	(18.0)	62.1	(18.5)	60.7	(18.6)	65.2	(18.0)	67.8	(18.1)	64.1	(19.0)
Fat intake [g/day]	61.4	(23.2)	55.0	(21.3)	56.0	(21.5)	55.1	(21.2)	55.9	(20.9)	54.3	(19.2)	48.3	(19.0)
Carbohydrate intake [g/day]	232.5	(68.3)	215.2	(67.2)	224.6	(64.6)	221.4	(63.7)	230.2	(58.9)	238.5	(58.6)	238.0	(60.9)
Food quotient	0.88	(N/A)	0.88	(N/A)	0.88	(N/A)	0.88	(N/A)	0.88	(N/A)	0.89	(N/A)	0.89	(N/A)
pBMR [kJ/day]	4874	(N/A)	4905	(N/A)	4828	(N/A)	4777	(N/A)	4597	(N/A)	4325	(N/A)	3975	(N/A)
Calibrated EI/pBMR	1.66	(N/A)	1.52	(N/A)	1.60	(N/A)	1.60	(N/A)	1.71	(N/A)	1.85	(N/A)	1.91	(N/A)

EI, energy intake; N/A, not available; pBMR, predicted basal metabolic rate. Energy intake conversion factor:1 kJ = 0.239 kcal

Participants included all those who had completed the dietary record. All continuous values are presented as mean (standard deviation). Body mass index was calculated as body weight (kg) divided by height squared (m^2^)

**Fig. 1:**
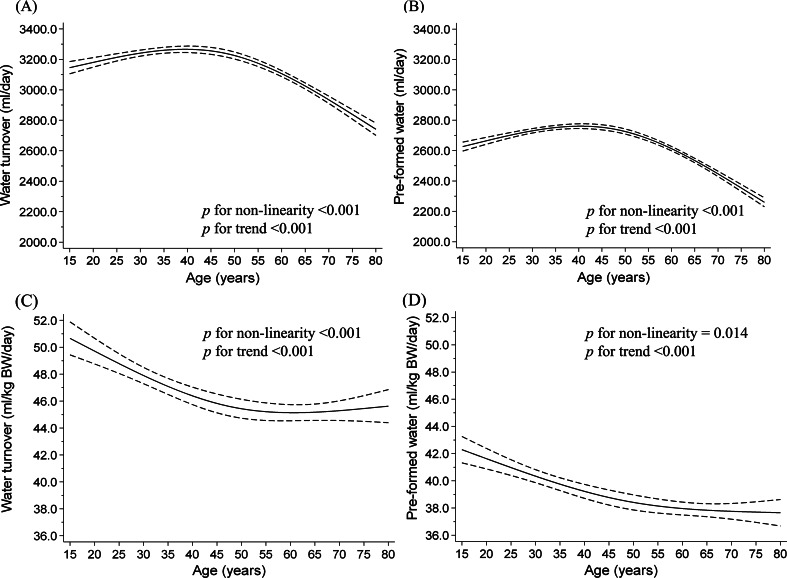
Relationship between water consumption and age among men illustrated by a restricted cubic spline model. (**A**) water turnover, (**B**) pre-formed water, (**C**) water turnover per kilogram body weight (BW), and (**D**) pre-formed water per kilogram BW. Solid lines represent mean water consumption, dashed lines represent 95% confidence intervals

**Fig. 2:**
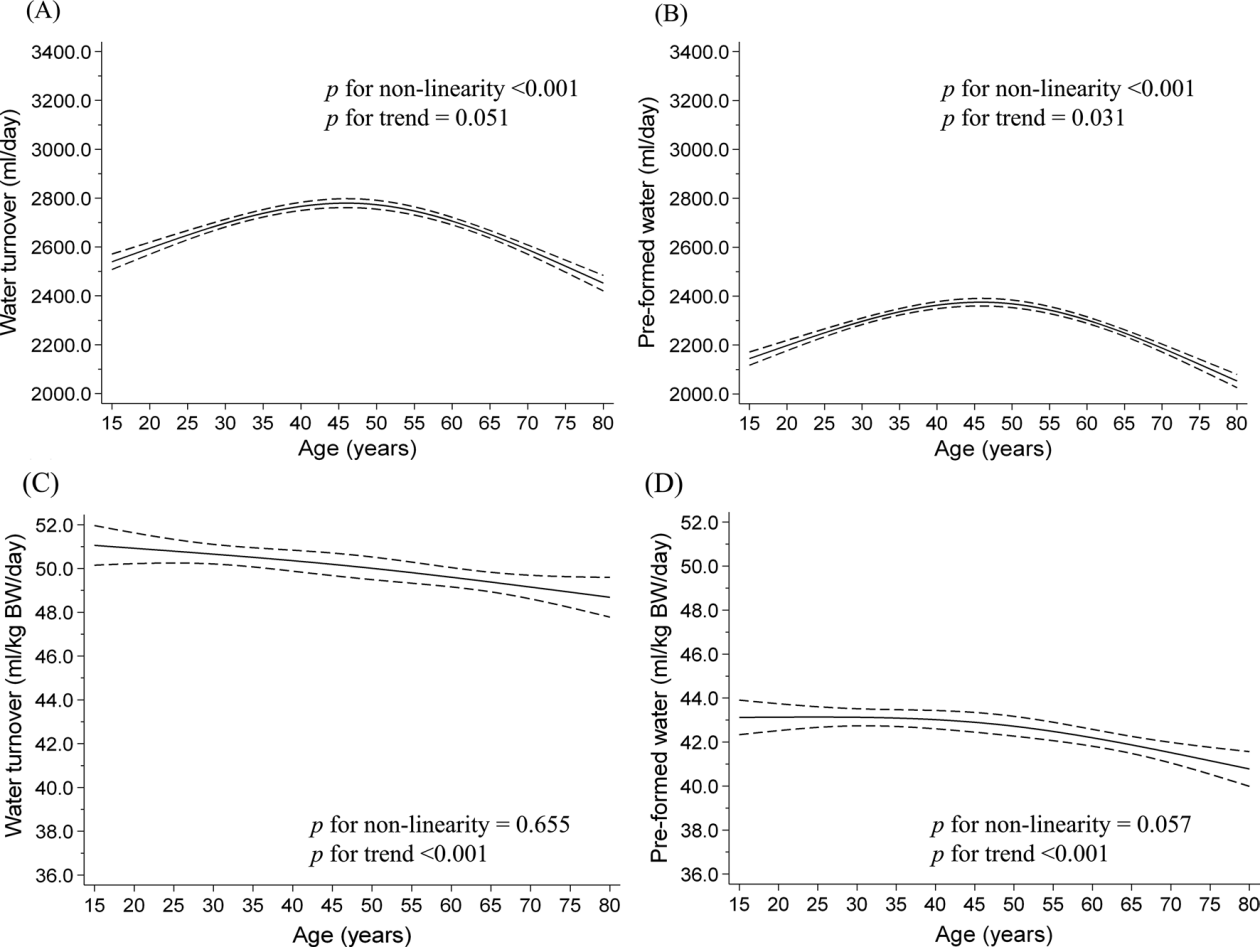
Relationship between water consumption and age by a restricted cubic spline model among women. (**A**) water turnover, (**B**) pre-formed water, (**C**) water turnover per kilogram body weight (BW), and (**D**) pre-formed water per kilogram BW. Solid lines represent mean water consumption, dashed lines represent 95% confidence intervals

Table 2 shows the distribution of water consumption according to age and sex estimated using the prediction equation. The average WT for the 15–19, 20–29, 30–39, 40–49, 50–59, 60–69, and ≥ 70 years was 3291, 3151, 3213, 3243, 3205, 3104, and 2790 ml/day, respectively in men, and 2641, 2594, 2741, 2739, 2753, 2707, and 2482 ml/day, respectively in women. Figures 1 and 2 show the dose-response relationship between water consumption and age using the restricted cubic spline model. WT and pre-formed water showed an inverse association with age in men ≥ 50 years (*p* for non-linearity < 0.001), whereas WT and pre-formed water per body weight showed a strong dose-dependent negative association with age ≤ 50 years; however, no significant differences were observed in older men (L-shaped relationship). Conversely, women showed a reverse U-shaped relationship between water consumption and age (*p* for non-linearity < 0.001), and a slightly inverse linear association of WT and pre-formed water per body weight with age (*p* for trend < 0.001). Although water consumption calculated using calibrated EI was underestimated from 2.1 to 9.2% compared to the values estimated using TEE in previous studies, relationships between age and water consumption were corroborated by the main and sensitivity analyses, except for WT per body weight in women (Supplementary Tables 2–3 and Supplementary Figs. 1–2). From the results of the main and sensitivity analyses, the ranges of WT and pre-formed water per body weight were 45–56 and 37–47 ml/day, respectively, in both sexes. In addition, water consumption estimated using uncalibrated EI was underestimated from 6.7 to 14.3% compared to values estimated using TEE in previous studies (Supplementary Tables 4–5).

**Table 2:** Distribution of water consumption according to age and sex as estimated by the prediction equation

	*n*	Water consumption estimated by prediction equation (ml/day)									
		Water turnover		Metabolic water		Respiratory water		Transcutaneous water		Pre-formed water	
**Men**											
15–19 years	559	3291	(3265 to 3315)	355	(N/A)	124	(N/A)	76	(N/A)	2735	(2712 to 2758)
20–29 years	710	3151	(3125 to 3176)	309	(N/A)	108	(N/A)	80	(N/A)	2654	(2628 to 2679)
30–39 years	1207	3213	(3204 to 3221)	307	(N/A)	107	(N/A)	81	(N/A)	2718	(2709 to 2726)
40–49 years	1581	3243	(3236 to 3250)	311	(N/A)	109	(N/A)	81	(N/A)	2742	(2734 to 2748)
50–59 years	1486	3205	(3183 to 3226)	316	(N/A)	110	(N/A)	80	(N/A)	2699	(2677 to 2720)
60–69 years	2307	3104	(3072 to 3135)	318	(N/A)	111	(N/A)	78	(N/A)	2597	(2565 to 2628)
≥ 70 years	2696	2790	(2746 to 2833)	295	(N/A)	103	(N/A)	75	(N/A)	2318	(2275 to 2360)
**Women**											
15–19 years	491	2641	(2606 to 2676)	258	(N/A)	90	(N/A)	66	(N/A)	2228	(2193 to 2262)
20–29 years	779	2594	(2566 to 2621)	237	(N/A)	83	(N/A)	68	(N/A)	2206	(2179 to 2233)
30–39 years	1350	2741	(2729 to 2753)	247	(N/A)	86	(N/A)	68	(N/A)	2339	(2328 to 2351)
40–49 years	1819	2739	(2735 to 2743)	245	(N/A)	86	(N/A)	69	(N/A)	2339	(2335 to 2343)
50–59 years	1777	2753	(2736 to 2771)	252	(N/A)	88	(N/A)	69	(N/A)	2345	(2327 to 2363)
60–69 years	2641	2707	(2684 to 2729)	256	(N/A)	90	(N/A)	67	(N/A)	2294	(2272 to 2316)
≥ 70 years	3498	2482	(2452 to 2512)	244	(N/A)	86	(N/A)	64	(N/A)	2088	(2058 to 2117)

N/A, not available

The survey was conducted between October and November 2016. The mean temperature and relative humidity during the survey period were 10.9℃ and 74.7%, respectively. The values are presented as mean (95% confidence interval)

We compared the distribution of pre-formed water estimated by the prediction equation and DRs according to sex- and age-stratified models (Table 3). Pre-formed-water estimated by DR was underestimated by approximately 20 to 40% compared with those estimated by prediction equation in both sexes. These underestimation levels were negatively associated with age (*p* for trend < 0.001). Similar results were obtained from the sensitivity analysis (Supplementary Table 6).

**Table 3:** Comparison of distribution of pre-formed water estimated by the prediction equation and dietary record according to a sex and age-stratified model

	*n*	Pre-formed water (ml/day)					Difference			
		Equation ^a^		Dietary record ^b^			Absolute (ml/day)		Relative (%)	
		Mean	95% CI	Mean	95% CI		Mean	95% CI	Mean	95% CI
**Men**										
15–19 years	559	2735	(2712 to 2758)	1648	(N/A)		-1087	(-1111 to -1064)	-39.7	(-40.3 to -39.2)
20–29 years	710	2654	(2628 to 2679)	1620	(N/A)		-1034	(-1059 to -1008)	-38.9	(-39.5 to -38.4)
30–39 years	1207	2718	(2709 to 2726)	1687	(N/A)		-1031	(-1040 to -1022)	-37.9	(-38.1 to -37.7)
40–49 years	1581	2742	(2734 to 2748)	1775	(N/A)		-967	(-974 to -960)	-35.3	(-35.4 to -35.1)
50–59 years	1486	2699	(2677 to 2720)	1867	(N/A)		-833	(-854 to -811)	-30.8	(-31.4 to -30.3)
60–69 years	2307	2597	(2565 to 2628)	1945	(N/A)		-652	(-684 to -621)	-25.1	(-26.0 to -24.2)
≥ 70 years	2696	2318	(2275 to 2360)	1802	(N/A)		-516	(-559 to -474)	-22.2	(-23.7 to -20.8)
*p* for trend ^c^							< 0.001		< 0.001	
**Women**										
15–19 years	491	2228	(2193 to 2262)	1299	(N/A)		-929	(-963 to -894)	-41.7	(-42.6 to -40.8)
20–29 years	779	2206	(2179 to 2233)	1312	(N/A)		-894	(-921 to -866)	-40.5	(-41.2 to -39.8)
30–39 years	1350	2339	(2328 to 2351)	1443	(N/A)		-896	(-908 to -884)	-38.3	(-38.6 to -38.0)
40–49 years	1819	2339	(2335 to 2343)	1494	(N/A)		-845	(-849 to -841)	-36.1	(-36.3 to -36.0)
50–59 years	1777	2345	(2327 to 2363)	1620	(N/A)		-725	(-742 to -707)	-30.9	(-31.4 to -30.4)
60–69 years	2641	2294	(2272 to 2316)	1680	(N/A)		-615	(-637 to -592)	-26.8	(-27.5 to -26.1)
≥ 70 years	3498	2088	(2058 to 2117)	1564	(N/A)		-524	(-554 to -494)	-25.1	(-26.1 to -24.0)
*p* for trend ^c^							< 0.001		< 0.001	

CI, confidence interval; N/A, not available

^a^Pre-formed water was calculated by a prediction equation using values of calibrated energy intake estimated from dietary records

^b^Since water intake from food estimated by dietary records has not been reported, the mean ratio of water in food in the dietary records of previous studies, 69%, was considered. The estimate of pre-formed water was calculated from the sum of water intake from food and drinks

^c^Linear trend p values were calculated for the regression model using the continuous variable of age

## Discussion

This study indicated the distribution of WT across age and sex in Japanese adolescents and adults, wherein WT was estimated using a prediction equation. WT and pre-formed water showed an inverse association with age in men aged ≥ 50 years, whereas a reverse U-shaped relationship between water consumption and age was observed in women. These results differed with adjustment for body weight. Pre-formed water estimated from DRs was approximately 20 to 40% underestimated compared with those estimated by the prediction equation in both sexes, and this underestimated amount was negatively associated with age. This is the first study to standardise values of water consumption in a Japanese population using a prediction equation and national large-scale survey data. These findings may be useful in setting water requirements for future dietary guidelines for the Japanese.

As previously stated, our results predict a different relationship of WT and age in men and women. The international DLW database group indicated that WT is greatest between the ages of 20–30 years in men and 25–60 years in women, and is lower in > 40 years men and > 65 years women [4]. Moreover, a previous study of American adults aged 40–79 years living in temperate climates reported that WT was inversely associated with age in both men and women [9]. Our findings are consistent with those of previous studies. These results suggest that age-related changes in WT in men aged > 50 years may be explained by changes in body weight because WT per body weight showed no significant differences with age after 50 years. In the 1998 National Nutrition Survey of Japan, 43.7% of all young Japanese women aged 15–39 indicated a desire to be ‘lean’ or ‘underweight’ [36], and the data we used also showed a reverse U-shaped relationship between body weight and age. This suggests that the low body weight of young women might contribute to the reverse U-shaped relationship between WT and age in women because similar findings were not observed in the results of WT per body weight. Considering that the logarithmic plots of WT and body weight in other mammals and humans are nearly linear [1], the target value for WT per body weight may also be applicable to individuals of different body masses living in similar environments. In environments with high external temperatures, fluid loss due to sweating increases, which increases daily water requirements [4, 5]. A previous study that used DRs over four days in each season to estimate the total water intake from food and drink (pre-formed water) observed higher values in the hot summer season than those in the winter [37]. The average annual temperature was 15.1ºC in Japan in 2012 [38], and the season in which the NHNS-J data were collected was closer to the average annual value than other seasons, suggesting that the autumn estimation may have reflected average annual water consumption better. Therefore, this study has the potential to provide standard values of average annual water consumption that can be used as targets when creating dietary guidelines applicable to public health policy aimed at avoiding inadequate water consumption.

Compared to the prediction equation, our findings indicated that DRs underestimated pre-formed water by approximately 20–40%. This is similar to the results of a previous study that underestimated water consumption estimated from self-report dietary assessment methods such as DRs and 24-hour dietary recalls compared with water consumption measured from biomarkers [11]. In addition, we showed that these underestimation levels were negatively correlated with age. Data from the 2012 NHNS-J indicated that the under-reporting of EI is inversely associated with age, and these findings are consistent with ours [14]. However, under-reporting of EI was associated with older age in the National Health and Nutrition Examination Survey in the United States [13]. Therefore, the relationship between the underestimation of pre-formed water estimated by dietary assessment and age needs to be examined in each country and region. These findings corroborate the fact that it is difficult to accurately evaluate self-reported dietary intake due to systematic errors associated with subject characteristics [13–15, 39]. Further research is needed on whether predictive equations can be used to accurately estimate WT and pre-formed water intake by age and sex, as an alternative to DRs. It has been previously reported that a biomarker calibration approach for self-reported EI, as opposed to uncalibrated EI, is strongly associated with mortality [30] and the prevalence of frailty [31]. The guidelines for nutritional epidemiological studies recommend the use of biomarkers in the assessment of dietary intake [40]. Thus, our results may underscore the importance of estimating dietary intake using predictive equations that incorporate biomarkers. Using a prediction equation to estimate pre-formed water can partially solve the problem of systematic errors that have hindered nutritional epidemiological studies for decades, thereby bridging the gap in results from different studies [41].

This study has the advantage of using data from participants selected by stratified random sampling across 47 prefectures in Japan. Additionally, the sample size used to evaluate the distribution of water consumption in this study may be sufficient to provide strong support for our findings. However, there are some methodological limitations. First, self-reported data for dietary assessment may have been affected by systematic errors related to individuals’ characteristics, such as age and BMI [13–15, 39], which hinders the accurate evaluation of dietary intake. In addition, they may not reflect an individual’s habitual dietary intake because the DR was maintained for only one day [25]. Consequently, we may have underestimated WT due to the underestimation of EI in our study, in which EI/BMR was assumed to be PAL. Although the ratio of underestimation of EI from DRs to TEE measured by the DLW method in a previous study was corrected, it is possible that these relationships are not uniform across age groups. However, our results were similar after sensitivity analysis using data from previous Japanese studies that measured TEE using DLW methods. Second, we were unable to access any more data on the participants in this survey because we obtained all data from electronically available aggregated reports on the official website. In this context, the data used in the form of aggregated reports included the age category of 15–19 years. The equation used to predict WT was developed for people over 18 years of age, so there may have been systematic bias in this data that included people under 18 years of age. Moreover, it was not possible to calculate standard deviations or 95% CIs for some variables. Thus, our findings need to be re-evaluated using original data or other secondary databases such as National Health and Nutrition Examination Survey because many indicators in the equation may deviate significantly from the actual situation by using unified conjectured data as personal data could not be obtained. Third, although we used the data of participants selected by stratified random sampling, only 44.4% underwent the 2016 NHNS-J. Thus, these participants may have been more health-aware than the general Japanese population, opening our study to the possibility of selection bias. Fourth, owing to its cross-sectional design, this study is limited to describing population-level changes. Thus, it cannot be extended to understand how a specific individual’s water consumption changes with age [42]. Considering these limitations, extrapolation to participants with attributes and environmental conditions other than those included in this study should be performed with caution. Therefore, further longitudinal studies with repeated measurements and randomly sampled participants are needed to evaluate longitudinal changes in water consumption over time according to age and sex.

## Conclusions

We determined the standard values of water consumption in the Japanese population using a predictive equation and national large-scale survey data. Our presented methodological approach may help assess water requirements in other regions or countries by using national nutrition survey data and prediction equations and is useful for setting target values for future dietary reference intakes. This method needs to be corrected for the underestimation of EI estimated by self-reported dietary assessment for a more accurate estimate of the standard value of WT.

### Electronic supplementary material

Below is the link to the electronic supplementary material.

**Supplementary Material 1**: **Supplementary Table 1.** Difference in energy intake assessed by dietary record and previous total energy expenditure data measured by the doubly labelled water method. **Supplementary Table 2.** Comparison of distribution of water turnover estimated by a prediction equation using calibrated energy intake assessed by dietary record with previous total energy expenditure data measured by the doubly labelled water method according to a sex- and age-stratified model. **Supplementary Table 3.** Comparison of distribution of pre-formed water estimated by prediction equation using calibrated energy intake assessed by dietary record with previous total energy expenditure data measured by the doubly labelled water method according to a sex- and age-stratified model. **Supplementary Table 4.** Comparison of distribution of water turnover estimated by prediction equation using uncalibrated energy intake assessed by dietary record with previous total energy expenditure data measured by the doubly labelled water method according to a sex- and age-stratified model. **Supplementary Table 5.** Comparison of distribution of pre-formed water estimated by prediction equation using uncalibrated energy intake assessed by dietary record with previous total energy expenditure data measured by the doubly labelled water method according to a sex- and age-stratified model. **Supplementary Table 6.** Results of sensitivity analysis for comparison of distribution of pre-formed water estimated by a prediction equation and dietary record according to a sex- and agestratified model. **Supplementary Figure 1.** Results of sensitivity analysis for the relationship between water consumption and age among men illustrated by a restricted cubic spline model. **Supplementary Figure 2.** Results of sensitivity analysis for the relationship between water consumption and age by a restricted cubic spline model among women.

## Data Availability

Annual reports and summary tables of the National Health and Nutrition Survey, Japan are published on the official website of the survey by the Ministry of Health, Labour and Welfare. Results are published each year in the form of electronically available aggregated data [http://www.mhlw.go.jp/bunya/kenkou/kenkou_eiyou_chousa.html]. Moreover, individual-level data from the National Health and Nutrition Survey, Japan are electronically available for scientific research if approved by the Ministry of Health, Labour and Welfare through official application procedures under Article 33 of the Statistics Act; however, currently, these data are only available in Japanese.
