# Supplementary material for: Distribution of water turnover by sex and age as estimated by prediction equation in Japanese adolescents and adults: the 2016 National Health and Nutrition Survey, Japan
Source: Nutr J. 2023 Nov 29;22:64. doi: 10.1186/s12937-023-00896-z (PMC10685525; doi:10.1186/s12937-023-00896-z)
Supplement: Supplementary file 1 — Supplementary Material 1: Supplementary Table 1. Difference in energy intake assessed by dietary record and previous total energy expenditure data measured by the doubly labelled water method. Supplementary Table 2. Comparison of distribution of water turnover estimated by a prediction equation using calibrated energy intake assessed by dietary record with previous total energy expenditure data measured by the doubly labelled water method according to a sex- and age-stratified model. Supplementary Table 3. Comparison of distribution of pre-formed water estimated by prediction equation using calibrated energy intake assessed by dietary record with previous total energy expenditure data measured by the doubly labelled water method according to a sex- and age-stratified model. Supplementary Table 4. Comparison of distribution of water turnover estimated by prediction equation using uncalibrated energy intake assessed by dietary record with previous total energy expenditure data measured by the doubly labelled water method according to a sex- and age-stratified model. Supplementary Table 5. Comparison of distribution of pre-formed water estimated by prediction equation using uncalibrated energy intake assessed by dietary record with previous total energy expenditure data measured by the doubly labelled water method according to a sex- and age-stratified model. Supplementary Table 6. Results of sensitivity analysis for comparison of distribution of pre-formed water estimated by a prediction equation and dietary record according to a sex- and agestratified model. Supplementary Figure 1. Results of sensitivity analysis for the relationship between water consumption and age among men illustrated by a restricted cubic spline model. Supplementary Figure 2. Results of sensitivity analysis for the relationship between water consumption and age by a restricted cubic spline model among women. [file 12937_2023_896_MOESM1_ESM.docx]

**Supplemental Data**

“Distribution of water turnover by sex and age as estimated by prediction equation in Japanese adolescents and adults: The 2016 National Health and Nutrition Survey, Japan”

Authors: Daiki Watanabe, Yumiko Inoue, Motohiko Miyachi

**SUPPLEMENTARY TABLES AND FIGURE**

**Supplementary Table 1.** Difference in energy intake assessed by dietary record and previous total energy expenditure data measured by the doubly labelled water method.

**Supplementary Table 2.** Comparison of distribution of water turnover estimated by a prediction equation using calibrated energy intake assessed by dietary record with previous total energy expenditure data measured by the doubly labelled water method according to a sex- and age-stratified model.

**Supplementary Table 3.** Comparison of distribution of pre-formed water estimated by prediction equation using calibrated energy intake assessed by dietary record with previous total energy expenditure data measured by the doubly labelled water method according to a sex- and age-stratified model.

**Supplementary Table 4.** Comparison of distribution of water turnover estimated by prediction equation using uncalibrated energy intake assessed by dietary record with previous total energy expenditure data measured by the doubly labelled water method according to a sex- and age-stratified model.

**Supplementary Table 5.** Comparison of distribution of pre-formed water estimated by prediction equation using uncalibrated energy intake assessed by dietary record with previous total energy expenditure data measured by the doubly labelled water method according to a sex- and age-stratified model.

**Supplementary Table 6.** Results of sensitivity analysis for comparison of distribution of pre-formed water estimated by a prediction equation and dietary record according to a sex- and age-stratified model.

**Supplementary Figure 1.** Results of sensitivity analysis for the relationship between water consumption and age among men illustrated by a restricted cubic spline model.

**Supplementary Figure 2.** Results of sensitivity analysis for the relationship between water consumption and age by a restricted cubic spline model among women.

**Supplementary Table 1** Difference in energy intake assessed by dietary record and previous total energy expenditure data measured by the doubly labelled water method

|  | Participants by age group | | | | | | | | | | | | | |
| --- | --- | --- | --- | --- | --- | --- | --- | --- | --- | --- | --- | --- | --- | --- |
|  | 15-19 years | | 20-29 years | | 30-39 years | | 40-49 years | | 50-59 years | | 60-69 years | | ≥70 years | |
| **Men, *n*** | 559 | | 710 | | 1207 | | 1581 | | 1486 | | 2307 | | 2696 | |
| EI [kJ/day] | 10148 | (2678) | 8842 | (2824) | 8754 | (2477) | 8877 | (2356) | 8976 | (2188) | 9037 | (2125) | 8353 | (1941) |
| Calibrated EI [kJ/day] | 11061 | (2916) | 9638 | (3075) | 9541 | (2699) | 9676 | (2569) | 9784 | (2385) | 9850 | (2318) | 9105 | (2117) |
| TEE [kJ/day] ^a^ | 12400 | (2400) | 11010 | (1560) | 11110 | (2200) | 10800 | (1520) | 10230 | (1300) | 10810 | (2110) | 9908 | (1799) |
| pBMR [kJ/day] | 6237 | (N/A) | 6496 | (N/A) | 6439 | (N/A) | 6380 | (N/A) | 6147 | (N/A) | 5801 | (N/A) | 5368 | (N/A) |
| EI/pBMR | 1.63 | (N/A) | 1.36 | (N/A) | 1.36 | (N/A) | 1.39 | (N/A) | 1.46 | (N/A) | 1.56 | (N/A) | 1.56 | (N/A) |
| Calibrated EI/pBMR | 1.77 | (N/A) | 1.48 | (N/A) | 1.48 | (N/A) | 1.52 | (N/A) | 1.59 | (N/A) | 1.70 | (N/A) | 1.70 | (N/A) |
| PAL ^b^ | 1.99 | (N/A) | 1.69 | (N/A) | 1.72 | (N/A) | 1.69 | (N/A) | 1.66 | (N/A) | 1.86 | (N/A) | 1.85 | (N/A) |
| Difference of EI-TEE [kJ/day] ^c^ | -2244 | (-18.1) | -2161 | (-19.6) | -2349 | (-21.2) | -1916 | (-17.8) | -1247 | (-12.2) | -1766 | (-16.4) | -1554 | (-15.7) |
| Difference of EI/pBMR-PAL ^c^ | -0.36 | (-18.1) | -0.33 | (-19.6) | -0.36 | (-21.2) | -0.30 | (-17.8) | -0.20 | (-12.2) | -0.30 | (-16.4) | -0.29 | (-15.7) |
| Difference of CEI-TEE [kJ/day] ^c^ | -1331 | (-10.7) | -1365 | (-12.4) | -1561 | (-14.1) | -1117 | (-10.4) | -440 | (-4.3) | -953 | (-8.8) | -803 | (-8.1) |
| Difference of CEI/pBMR-PAL ^c^ | -0.21 | (-10.7) | -0.21 | (-12.4) | -0.24 | (-14.1) | -0.18 | (-10.4) | -0.07 | (-4.3) | -0.16 | (-8.8) | -0.15 | (-8.1) |
| **Women, *n*** | 491 | | 779 | | 1350 | | 1819 | | 1777 | | 2641 | | 3498 | |
| EI [kJ/day] | 7420 | (2033) | 6823 | (1895) | 7087 | (1866) | 7017 | (1828) | 7225 | (1669) | 7324 | (1615) | 6957 | (1699) |
| Calibrated EI [kJ/day] | 8087 | (2218) | 7437 | (2067) | 7725 | (2033) | 7649 | (1992) | 7875 | (1820) | 7984 | (1761) | 7583 | (1849) |
| TEE [kJ/day] ^a^ | 9300 | (1600) | 8290 | (1510) | 8530 | (1650) | 8400 | (980) | 8170 | (920) | 8530 | (1420) | 8180 | (1188) |
| pBMR [kJ/day] | 4874 | (N/A) | 4905 | (N/A) | 4828 | (N/A) | 4777 | (N/A) | 4597 | (N/A) | 4325 | (N/A) | 3975 | (N/A) |
| EI/pBMR | 1.52 | (N/A) | 1.39 | (N/A) | 1.47 | (N/A) | 1.47 | (N/A) | 1.57 | (N/A) | 1.69 | (N/A) | 1.75 | (N/A) |
| Calibrated EI/pBMR | 1.66 | (N/A) | 1.52 | (N/A) | 1.60 | (N/A) | 1.60 | (N/A) | 1.71 | (N/A) | 1.85 | (N/A) | 1.91 | (N/A) |
| PAL ^b^ | 1.91 | (N/A) | 1.69 | (N/A) | 1.77 | (N/A) | 1.76 | (N/A) | 1.78 | (N/A) | 1.97 | (N/A) | 2.06 | (N/A) |
| Difference of EI-TEE [kJ/day] ^c^ | -1874 | (-20.2) | -1461 | (-17.6) | -1438 | (-16.9) | -1377 | (-16.4) | -940 | (-11.5) | -1200 | (-14.1) | -1222 | (-14.9) |
| Difference of EI/pBMR-PAL ^c^ | -0.38 | (-20.2) | -0.30 | (-17.6) | -0.30 | (-16.9) | -0.29 | (-16.4) | -0.20 | (-11.5) | -0.28 | (-14.1) | -0.31 | (-14.9) |
| Difference of CEI-TEE [kJ/day] ^c^ | -1207 | (-13.0) | -847 | (-10.2) | -800 | (-9.4) | -746 | (-8.9) | -290 | (-3.5) | -541 | (-6.3) | -596 | (-7.2) |
| Difference of CEI/pBMR-PAL ^c^ | -0.25 | (-13.0) | -0.17 | (-10.2) | -0.17 | (-9.4) | -0.16 | (-8.9) | -0.06 | (-3.5) | -0.12 | (-6.3) | -0.15 | (-7.2) |

CEI, calibrated energy intake; EI, energy intake; N/A, not available; PAL, physical activity level; pBMR, predicted basal metabolic rate; TEE, total energy expenditure. Energy intake conversion factor:1 kJ=0.239 kcal. The number of participants indicates the number who completed the dietary record.

All continuous values are shown as mean (standard deviation).

^a^ The data used are values from previous studies that reported TEE measured using the doubly labelled water method in a Japanese population.

^b^ PAL was calculated as TEE divided by the pBMR.

^c^ All continuous values are shown as the mean difference (relative difference [%]).

**Supplementary Table 2** Comparison of distribution of water turnover estimated by a prediction equation using calibrated energy intake assessed by dietary record with previous total energy expenditure data measured by the doubly labelled water method according to a sex- and age-stratified model

|  | *n* | Water turnover (ml/day) | | | |  | Difference | | | |
| --- | --- | --- | --- | --- | --- | --- | --- | --- | --- | --- |
|  |  | CEI of dietary record | | Previous data of TEE ^a^ | |  | Absolute (ml/day) | | Relative (%) | |
|  |  | Mean | 95% CI | Mean | 95% CI |  | Mean | 95% CI | Mean | 95% CI |
| **Men** |  |  | |  | |  |  |  |  |  |
| 15-19 years | 559 | 3291 | (3265 to 3315) | 3520 | (3499 to 3542) |  | -230 | (-236 to -224) | -6.5 | (-6.7 to -6.3) |
| 20-29 years | 710 | 3151 | (3125 to 3176) | 3377 | (3351 to 3403) |  | -226 | (-228 to -224) | -6.7 | (-6.8 to -6.6) |
| 30-39 years | 1207 | 3213 | (3204 to 3221) | 3474 | (3464 to 3483) |  | -261 | (-262 to -260) | -7.5 | (-7.6 to -7.4) |
| 40-49 years | 1581 | 3243 | (3236 to 3250) | 3432 | (3426 to 3438) |  | -188 | (-189 to -188) | -5.5 | (-5.6 to -5.4) |
| 50-59 years | 1486 | 3205 | (3183 to 3226) | 3282 | (3261 to 3303) |  | -77 | (-78 to -76) | -2.3 | (-2.4 to -2.2) |
| 60-69 years | 2307 | 3104 | (3072 to 3135) | 3281 | (3251 to 3311) |  | -177 | (-179 to -175) | -5.4 | (-5.5 to -5.2) |
| ≥70 years | 2696 | 2790 | (2746 to 2833) | 2951 | (2911 to 2992) |  | -161 | (-165 to -158) | -5.5 | (-5.7 to -5.2) |
| *p* for trend ^b^ |  |  |  |  |  |  | <0.001 | | <0.001 | |
| **Women** |  |  |  |  |  |  |  |  |  |  |
| 15-19 years | 491 | 2641 | (2606 to 2676) | 2907 | (2872 to 2943) |  | -266 | (-270 to -262) | -9.2 | (-9.3 to -9.0) |
| 20-29 years | 779 | 2594 | (2566 to 2621) | 2779 | (2751 to 2808) |  | -186 | (-188 to -183) | -6.7 | (-6.8 to -6.6) |
| 30-39 years | 1350 | 2741 | (2729 to 2753) | 2919 | (2906 to 2932) |  | -178 | (-179 to -177) | -6.1 | (-6.2 to -6.0) |
| 40-49 years | 1819 | 2739 | (2735 to 2743) | 2907 | (2904 to 2910) |  | -168 | (-169 to -166) | -5.8 | (-5.9 to -5.7) |
| 50-59 years | 1777 | 2753 | (2736 to 2771) | 2821 | (2804 to 2839) |  | -68 | (-69 to -67) | -2.4 | (-2.5 to -2.3) |
| 60-69 years | 2641 | 2707 | (2684 to 2729) | 2841 | (2821 to 2862) |  | -135 | (-136 to -132) | -4.7 | (-4.8 to -4.6) |
| ≥70 years | 3498 | 2482 | (2452 to 2512) | 2643 | (2616 to 2671) |  | -162 | (-165 to -157) | -6.1 | (-6.2 to -5.9) |
| *p* for trend ^b^ |  |  |  |  |  |  | <0.001 | | <0.001 | |

CEI, calibrated energy intake; CI, confidence interval; TEE, total energy expenditure.

^a^ The data used are values from previous studies that reported TEE measured using the doubly labelled water method in a Japanese population.

^b^ Linear trend p values were calculated with the regression model using continuous variables of age.

**Supplementary Table 3** Comparison of distribution of pre-formed water estimated by prediction equation using calibrated energy intake assessed by dietary record with previous total energy expenditure data measured by the doubly labelled water method according to a sex- and age-stratified model

|  | *n* | Pre-formed water (ml/day) | | | |  | Difference | | | |
| --- | --- | --- | --- | --- | --- | --- | --- | --- | --- | --- |
|  |  | CEI of dietary record | | Previous data of TEE ^a^ | |  | Absolute (ml/day) | | Relative (%) | |
|  |  | Mean | 95% CI | Mean | 95% CI |  | Mean | 95% CI | Mean | 95% CI |
| **Men** |  |  | |  | |  |  |  |  |  |
| 15-19 years | 559 | 2735 | (2712 to 2758) | 2908 | (2886 to 2928) |  | -172 | (-178 to -166) | -5.9 | (-6.1 to -5.6) |
| 20-29 years | 710 | 2654 | (2628 to 2679) | 2821 | (2795 to 2847) |  | -167 | (-169 to -164) | -5.9 | (-6.0 to -5.8) |
| 30-39 years | 1207 | 2718 | (2709 to 2726) | 2912 | (2901 to 2921) |  | -193 | (-194 to -192) | -6.6 | (-6.7 to -6.5) |
| 40-49 years | 1581 | 2742 | (2734 to 2748) | 2881 | (2875 to 2887) |  | -140 | (-141 to -139) | -4.9 | (-5.0 to -4.8) |
| 50-59 years | 1486 | 2699 | (2677 to 2720) | 2757 | (2735 to 2778) |  | -58 | (-59 to -57) | -2.1 | (-2.2 to -2.0) |
| 60-69 years | 2307 | 2597 | (2565 to 2628) | 2732 | (2702 to 2761) |  | -135 | (-137 to -133) | -5.0 | (-5.1 to -4.8) |
| ≥70 years | 2696 | 2318 | (2275 to 2360) | 2444 | (2404 to 2483) |  | -126 | (-129 to -122) | -5.2 | (-5.3 to -4.9) |
| *p* for trend ^b^ |  |  |  |  |  |  | <0.001 | | <0.001 | |
| **Women** |  |  |  |  |  |  |  |  |  |  |
| 15-19 years | 491 | 2228 | (2193 to 2262) | 2442 | (2407 to 2477) |  | -215 | (-218 to -210) | -8.8 | (-8.9 to -8.6) |
| 20-29 years | 779 | 2206 | (2179 to 2233) | 2356 | (2326 to 2385) |  | -149 | (-151 to -147) | -6.4 | (-6.5 to -6.2) |
| 30-39 years | 1350 | 2339 | (2328 to 2351) | 2483 | (2470 to 2496) |  | -144 | (-145 to -142) | -5.8 | (-5.9 to -5.7) |
| 40-49 years | 1819 | 2339 | (2335 to 2343) | 2475 | (2472 to 2478) |  | -136 | (-137 to -134) | -5.5 | (-5.6 to -5.4) |
| 50-59 years | 1777 | 2345 | (2327 to 2363) | 2400 | (2383 to 2417) |  | -55 | (-56 to -54) | -2.3 | (-2.4 to -2.2) |
| 60-69 years | 2641 | 2294 | (2272 to 2316) | 2406 | (2385 to 2426) |  | -111 | (-113 to -109) | -4.6 | (-4.7 to -4.4) |
| ≥70 years | 3498 | 2088 | (2058 to 2117) | 2223 | (2196 to 2250) |  | -136 | (-139 to -131) | -6.1 | (-6.3 to -5.8) |
| *p* for trend ^b^ |  |  |  |  |  |  | <0.001 | | <0.001 | |

CEI, calibrated energy intake; CI, confidence interval; TEE, total energy expenditure.

^a^ The data used are values from previous studies that reported TEE measured using the doubly labelled water method in a Japanese population.

^b^ Linear trend p values were calculated with the regression model using continuous variables of age.

**Supplementary Table 4** Comparison of distribution of water turnover estimated by prediction equation using uncalibrated energy intake assessed by dietary record with previous total energy expenditure data measured by the doubly labelled water method according to a sex- and age-stratified model

|  | *n* | Water turnover (ml/day) | | | |  | Difference | | | |
| --- | --- | --- | --- | --- | --- | --- | --- | --- | --- | --- |
|  |  | EI of dietary record | | Previous data of TEE ^a^ | |  | Absolute (ml/day) | | Relative (%) | |
|  |  | Mean | 95% CI | Mean | 95% CI |  | Mean | 95% CI | Mean | 95% CI |
| **Men** |  |  | |  | |  |  |  |  |  |
| 15-19 years | 559 | 3133 | (3105 to 3160) | 3520 | (3499 to 3542) |  | -387 | (-397 to -377) | -11.0 | (-11.3 to -10.6) |
| 20-29 years | 710 | 3019 | (2993 to 3045) | 3377 | (3351 to 3403) |  | -358 | (-361 to -354) | -10.6 | (-10.7 to -10.4) |
| 30-39 years | 1207 | 3081 | (3073 to 3088) | 3474 | (3464 to 3483) |  | -393 | (-394 to -390) | -11.3 | (-11.4 to -11.2) |
| 40-49 years | 1581 | 3109 | (3100 to 3116) | 3432 | (3426 to 3438) |  | -323 | (-324 to -321) | -9.4 | (-9.5 to -9.3) |
| 50-59 years | 1486 | 3064 | (3041 to 3086) | 3282 | (3261 to 3303) |  | -218 | (-219 to -217) | -6.7 | (-6.8 to -6.5) |
| 60-69 years | 2307 | 2953 | (2920 to 2986) | 3281 | (3251 to 3311) |  | -328 | (-331 to -324) | -10.0 | (-10.2 to -9.7) |
| ≥70 years | 2696 | 2639 | (2592 to 2685) | 2951 | (2911 to 2992) |  | -312 | (-318 to -305) | -10.6 | (-10.9 to -10.2) |
| *p* for trend ^b^ |  |  |  |  |  |  | <0.001 | | <0.001 | |
| **Women** |  |  |  |  |  |  |  |  |  |  |
| 15-19 years | 491 | 2494 | (2459 to 2529) | 2907 | (2872 to 2943) |  | -414 | (-420 to -407) | -14.3 | (-14.5 to -14.0) |
| 20-29 years | 779 | 2459 | (2433 to 2485) | 2779 | (2751 to 2808) |  | -321 | (-324 to -316) | -11.5 | (-11.6 to -11.4) |
| 30-39 years | 1350 | 2599 | (2588 to 2610) | 2919 | (2906 to 2932) |  | -320 | (-322 to -318) | -10.9 | (-11.0 to -10.9) |
| 40-49 years | 1819 | 2596 | (2592 to 2601) | 2907 | (2904 to 2910) |  | -310 | (-312 to -308) | -10.7 | (-10.8 to -10.5) |
| 50-59 years | 1777 | 2601 | (2582 to 2620) | 2821 | (2804 to 2839) |  | -220 | (-221 to -218) | -7.8 | (-7.8 to -7.7) |
| 60-69 years | 2641 | 2543 | (2518 to 2567) | 2841 | (2821 to 2862) |  | -299 | (-303 to -294) | -10.5 | (-10.7 to -10.2) |
| ≥70 years | 3498 | 2312 | (2279 to 2345) | 2643 | (2616 to 2671) |  | -331 | (-339 to -322) | -12.5 | (-12.9 to -12.1) |
| *p* for trend ^b^ |  |  |  |  |  |  | <0.001 | | <0.001 | |

CI, confidence interval; EI, energy intake; TEE, total energy expenditure

^a^ The data used are values from previous studies that reported TEE measured using the doubly labelled water method in a Japanese population.

^b^ Linear trend p values were calculated with the regression model using continuous variables of age.

**Supplementary Table 5** Comparison of distribution of pre-formed water estimated by prediction equation using uncalibrated energy intake assessed by dietary record with previous total energy expenditure data measured by the doubly labelled water method according to a sex- and age-stratified model

|  | *n* | Pre-formed water (ml/day) | | | |  | Difference | | | |
| --- | --- | --- | --- | --- | --- | --- | --- | --- | --- | --- |
|  |  | EI of dietary record | | Previous data of TEE ^a^ | |  | Absolute (ml/day) | | Relative (%) | |
|  |  | Mean | 95% CI | Mean | 95% CI |  | Mean | 95% CI | Mean | 95% CI |
| **Men** |  |  | |  | |  |  |  |  |  |
| 15-19 years | 559 | 2617 | (2591 to 2643) | 2908 | (2886 to 2928) |  | -290 | (-300 to -279) | -10.0 | (-10.3 to -9.5) |
| 20-29 years | 710 | 2557 | (2531 to 2582) | 2821 | (2795 to 2847) |  | -264 | (-267 to -261) | -9.4 | (-9.6 to -9.2) |
| 30-39 years | 1207 | 2621 | (2612 to 2628) | 2912 | (2901 to 2921) |  | -291 | (-292 to -288) | -10.0 | (-10.1 to -9.9) |
| 40-49 years | 1581 | 2641 | (2633 to 2649) | 2881 | (2875 to 2887) |  | -240 | (-241 to -238) | -8.3 | (-8.4 to -8.2) |
| 50-59 years | 1486 | 2593 | (2570 to 2615) | 2757 | (2735 to 2778) |  | -164 | (-165 to -162) | -6.0 | (-6.1 to -5.8) |
| 60-69 years | 2307 | 2482 | (2448 to 2514) | 2732 | (2702 to 2761) |  | -251 | (-254 to -247) | -9.2 | (-9.4 to -8.9) |
| ≥70 years | 2696 | 2200 | (2154 to 2245) | 2444 | (2404 to 2483) |  | -244 | (-250 to -237) | -10.0 | (-10.4 to -9.5) |
| *p* for trend ^b^ |  |  |  |  |  |  | <0.001 | | <0.001 | |
| **Women** |  |  |  |  |  |  |  |  |  |  |
| 15-19 years | 491 | 2109 | (2074 to 2144) | 2442 | (2407 to 2477) |  | -333 | (-339 to -326) | -13.7 | (-13.9 to -13.3) |
| 20-29 years | 779 | 2098 | (2071 to 2124) | 2356 | (2326 to 2385) |  | -258 | (-261 to -253) | -11.0 | (-11.1 to -10.8) |
| 30-39 years | 1350 | 2225 | (2214 to 2236) | 2483 | (2470 to 2496) |  | -258 | (-260 to -256) | -10.4 | (-10.5 to -10.3) |
| 40-49 years | 1819 | 2224 | (2219 to 2229) | 2475 | (2472 to 2478) |  | -251 | (-252 to -248) | -10.1 | (-10.2 to -10.0) |
| 50-59 years | 1777 | 2221 | (2202 to 2239) | 2400 | (2383 to 2417) |  | -179 | (-180 to -178) | -7.5 | (-7.6 to -7.3) |
| 60-69 years | 2641 | 2159 | (2135 to 2183) | 2406 | (2385 to 2426) |  | -247 | (-251 to -242) | -10.2 | (-10.4 to -9.9) |
| ≥70 years | 3498 | 1945 | (1913 to 1977) | 2223 | (2196 to 2250) |  | -278 | (-286 to -269) | -12.5 | (-12.9 to -12.0) |
| *p* for trend ^b^ |  |  |  |  |  |  | <0.001 | | <0.001 | |

CI, confidence interval; EI, energy intake; TEE, total energy expenditure

^a^ The data used are values from previous studies that reported TEE measured using the doubly labelled water method in a Japanese population.

^b^ Linear trend p values were calculated with the regression model using continuous variables of age.

**Supplementary Table 6** Results of sensitivity analysis for comparison of distribution of pre-formed water estimated by a prediction equation and dietary record according to a sex- and age-stratified model

|  | *n* | Pre-formed water (ml/day) | | | |  | Difference | | | |
| --- | --- | --- | --- | --- | --- | --- | --- | --- | --- | --- |
|  |  | Equation ^a^ | | Dietary record ^b^ | |  | Absolute (ml/day) | | Relative (%) | |
|  |  | Mean | 95% CI | Mean | 95% CI |  | Mean | 95% CI | Mean | 95% CI |
| **Men** |  |  | |  | |  |  |  |  |  |
| 15-19 years | 559 | 2908 | (2886 to 2928) | 1648 | (N/A) |  | -1259 | (-1280 to -1239) | -43.3 | (-43.7 to -42.9) |
| 20-29 years | 710 | 2821 | (2795 to 2847) | 1620 | (N/A) |  | -1201 | (-1227 to -1175) | -42.6 | (-43.1 to -42.0) |
| 30-39 years | 1207 | 2912 | (2901 to 2921) | 1687 | (N/A) |  | -1224 | (-1234 to -1214) | -42.0 | (-42.2 to -41.9) |
| 40-49 years | 1581 | 2881 | (2875 to 2887) | 1775 | (N/A) |  | -1107 | (-1113 to -1100) | -38.4 | (-38.5 to -38.3) |
| 50-59 years | 1486 | 2757 | (2735 to 2778) | 1867 | (N/A) |  | -890 | (-911 to -869) | -32.3 | (-32.8 to -31.8) |
| 60-69 years | 2307 | 2732 | (2702 to 2761) | 1945 | (N/A) |  | -788 | (-817 to -758) | -28.8 | (-29.6 to -28.0) |
| ≥70 years | 2696 | 2444 | (2404 to 2483) | 1802 | (N/A) |  | -642 | (-682 to -602) | -26.2 | (-27.4 to -25.0) |
| *p* for trend ^c^ |  |  |  |  |  |  | <0.001 | | <0.001 | |
| **Women** |  |  |  |  |  |  |  |  |  |  |
| 15-19 years | 491 | 2442 | (2407 to 2477) | 1299 | (N/A) |  | -1143 | (-1178 to -1108) | -46.8 | (-47.6 to -46.0) |
| 20-29 years | 779 | 2356 | (2326 to 2385) | 1312 | (N/A) |  | -1043 | (-1072 to -1014) | -44.3 | (-45.0 to -43.6) |
| 30-39 years | 1350 | 2483 | (2470 to 2496) | 1443 | (N/A) |  | -1040 | (-1053 to -1027) | -41.9 | (-42.2 to -41.6) |
| 40-49 years | 1819 | 2475 | (2472 to 2478) | 1494 | (N/A) |  | -981 | (-984 to -978) | -39.6 | (-39.7 to -39.5) |
| 50-59 years | 1777 | 2400 | (2383 to 2417) | 1620 | (N/A) |  | -780 | (-797 to -763) | -32.5 | (-33.0 to -32.0) |
| 60-69 years | 2641 | 2406 | (2385 to 2426) | 1680 | (N/A) |  | -726 | (-746 to -705) | -30.2 | (-30.8 to -29.6) |
| ≥70 years | 3498 | 2223 | (2196 to 2250) | 1564 | (N/A) |  | -660 | (-687 to -632) | -29.6 | (-30.5 to -28.8) |
| *p* for trend ^c^ |  |  |  |  |  |  | <0.001 | | <0.001 | |

CI, confidence interval; N/A, not available

^a^ Pre-formed water estimated by the prediction equation used values from previous studies that reported total energy expenditure measured using the doubly labelled water method in a Japanese population.

^b^ Since water intake from food estimated by dietary record has not reported, the mean ratio of water in the foods in the dietary records of previous studies, that is 69%, was considered. The estimate of pre-formed water was calculated from the sum of water intake from food and drinks.

^c^ Linear trend p values were calculated with the regression model using the continuous variable of age.


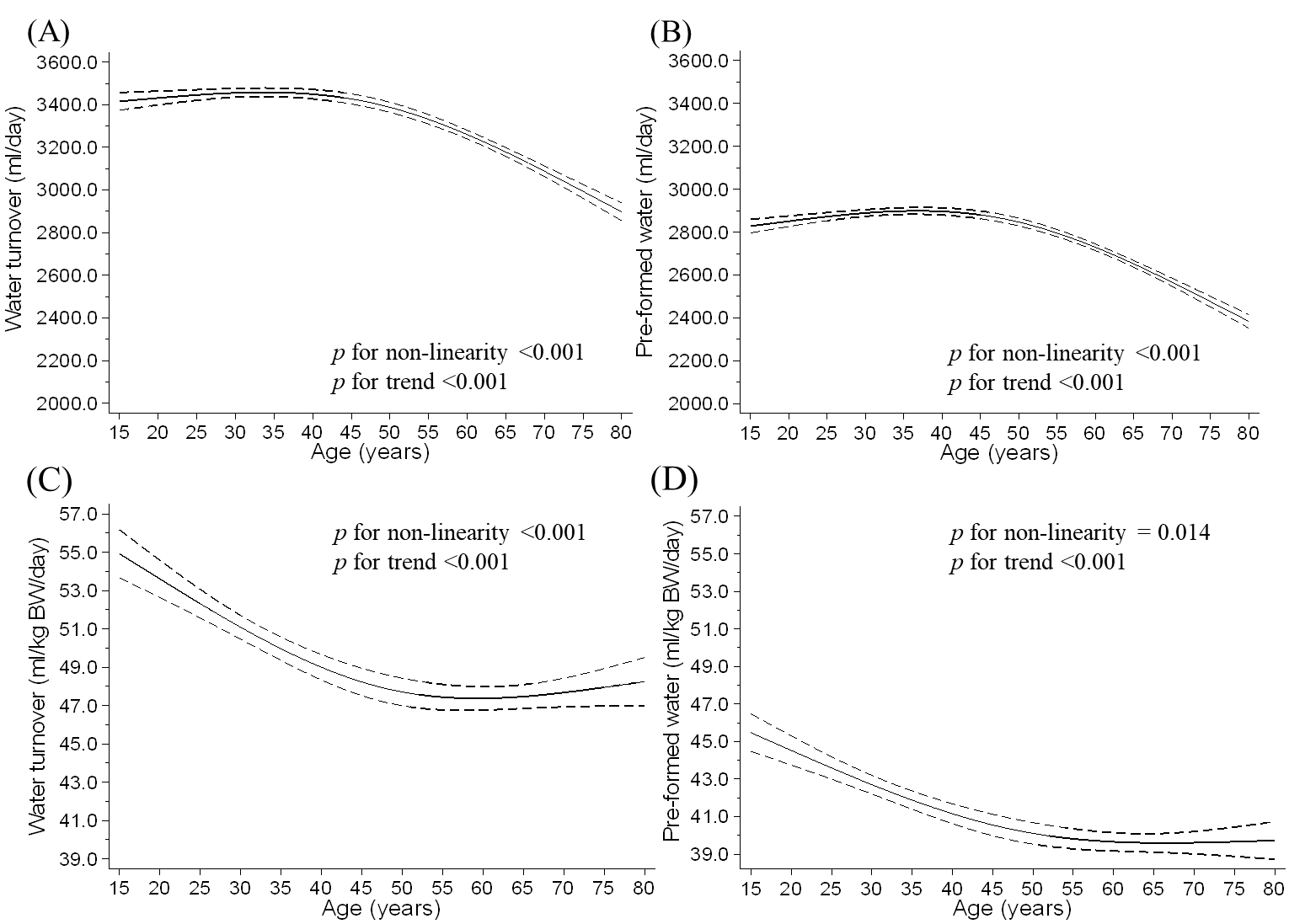


**Supplementary Figure 1** Results of sensitivity analysis for the relationship between water consumption and age among men illustrated by a restricted cubic spline model.

[A] water turnover, [B] pre-formed water, [C] water turnover per body weight (BW), and [D] pre-formed water per BW. Solid lines represent mean water consumption, dashed lines represent 95% confidence intervals.


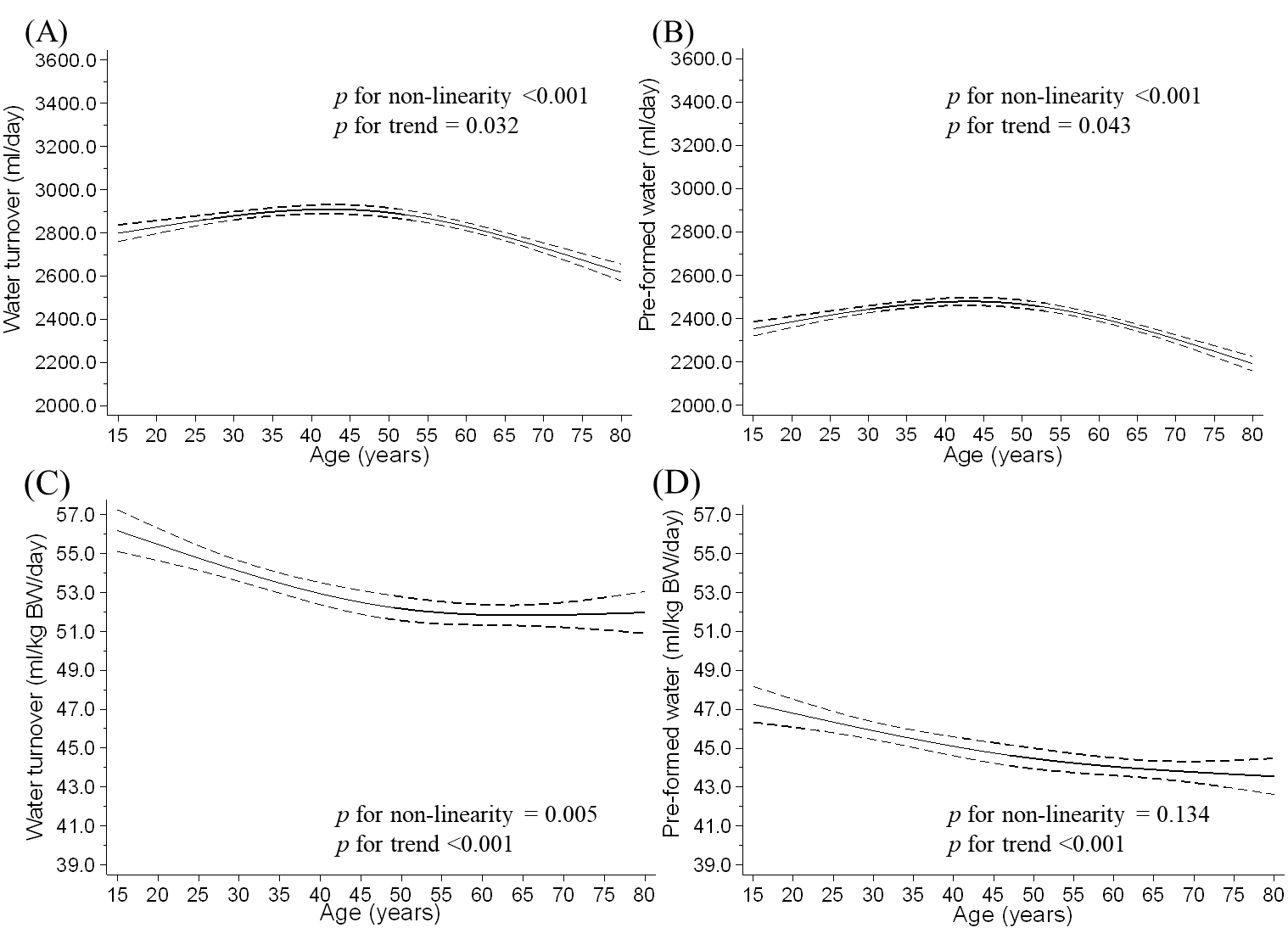


**Supplementary Figure 2** Results of sensitivity analysis for the relationship between water consumption and age by a restricted cubic spline model among women.

[A] water turnover, [B] pre-formed water, [C] water turnover per body weight (BW), and [D] pre-formed water per BW. Solid lines represent mean water consumption, dashed lines represent 95% confidence intervals.
